# High-voltage and long-lasting aqueous chlorine-ion battery by virtue of “water-in-salt” electrolyte

**DOI:** 10.1016/j.isci.2020.101976

**Published:** 2021-01-05

**Authors:** Tong Li, Mingqiang Li, Hang Li, Hu Zhao

**Affiliations:** 1 School of Energy and Power Engineering, Dalian University of Technology, Dalian 116024, China

**Keywords:** Electrochemical Energy Storage, Energy Materials, Energy Storage, Materials Characterization

## Abstract

Chloride-ion battery (CIB) is regarded as a promising electrochemical storage device due to their high theoretical volumetric capacities, low cost, and high abundance. However, low-cycle life limits its application in the energy storage field. Herein, we report a rechargeable CIB composed of a “water-in-salt” electrolyte, a zinc anode, and a carbon cathode (graphene, carbon nanotubes, carbon black). These cathodes exhibit initial reversible specific capacities of 136, 108, and 102 mAh g^−1^, respectively. Especially, a reversible discharge capacity of 95 mAh g^−1^ was retained after 2000 cycles when graphene is used as the cathode. Such high cycling stability was first reported in CIBs. Furthermore, the use of “water-in-salt” electrolytes has improved the discharge platform of aqueous CIBs to 2.6V. The charge and discharge mechanism of the carbon cathode was investigated by TEM, FTIR, Raman, and XPS, proving the chloride ions reversible absorption/desorption in carbon cathodes.

## Highlights

• Water-in-salt electrolytes expanded the electrochemical window of CIB into 3.1 V
• Reversible absorption/desorption of Cl^−^ ions into carbon cathodes
• Graphene cathode exhibits high capacity and superstable cycle life

## Graphical abstract

**Figure:**
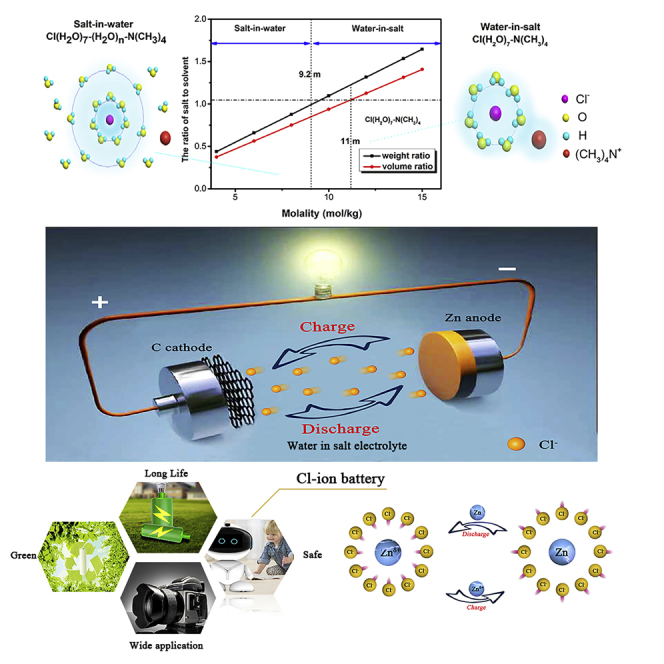

## Introduction

With the development of portable electronic devices, clean energy, and electric vehicles, there is a growing demand for rechargeable batteries with high cycle life, low cost, and high energy density that are environment-friendly and safe (Amine et al., 2014). Lithium-ion batteries dominate the present-day portable electronics market with their higher energy density, lower self-discharge, and good cycling stability (Zheng et al., 2006; Ding et al., 2018; Li et al., 2015). However, the practical implementation of Li-ion batteries on large-scale application is limited by its safety concerns, limited lithium resources, and high cost, leading to the intense exploration of alternative secondary batteries with safety, rich electrode material reserves, and low cost (Wessells et al., 2011; Ellis and Naazar, 2012; Li et al., 2018a, 2018b; Jayaprakash et al., 2011). Among various rechargeable ion batteries, chloride-ion battery (CIB) is regarded as the promising electrochemical systems due to their theoretical volumetric energy density (2,500 Wh/L) and abundant chloride-content for both electrolyte and electrode (Chen et al., 2019; Yin et al., 2019).

Xiangyu Zhao et al. firstly proposed the concept of rechargeable CIB composed of the metal chloride cathode, the metal anode (Li, Mg, Ca), and the binary ionic liquids electrolyte (Zhao et al., 2014). The issue is that the metal chloride cathodes can react with chloride ions in the ionic liquids electrolyte, leading to severe capacity decay of the CIB and thus deliver a poor cycle life. Metal oxychlorides (FeOCl, VOCl, Sb_4_O_5_Cl_2_) and chloride ion-doped conducting polymer materials such as PpyCl have been explored as cathode materials for CIBs (Zhao et al., 2013, 2017; Gao et al., 2014, 2016; Lakshmi et al., 2019). Although these new cathode materials show better stability and electrochemical performance than metal chlorides in ionic liquids electrolyte, the issue of electrode dissolution remains. Therefore, exploring highly reversible electrodes and compatible electrolytes are critical to the development of CIBs.

Carbon materials have been widely used in electrochemical energy storage systems due to their diversified structure, rich surface morphology, strong controllability, and excellent electrical conductivity (Zhang et al., 2015). In the field of rechargeable batteries, intercalation reactions and compounds of various graphite have been studied (Winter et al., 1998), for example, graphite anode has been used in ion batteries that can reversibly insert and extract cations (Slater et al., 2013; Wen et al., 2014). Recently, the co-intercalation of chloride ions and bromine ions in graphite cathode was reported; this makes it possible to realize the transfer of chloride ions in graphite (Yang et al., 2019), providing a new idea for the selection of the cathode of CIBs.

Rechargeable aqueous batteries are regarded as ideal choices for large-scale energy storage due to their high ionic conductivity, safety, low environmental impact, and low cost. However, the energy density and output voltage of such batteries are limited by the narrow electrochemical stability window of water (1.23 V) (Kim et al., 2014; Luo et al., 2010; Pan et al., 2016). Therefore, expanding the electrochemical stability window of aqueous electrolytes is the key to the development of aqueous rechargeable batteries. Recently, water-in-salt electrolytes widened the electrochemical window of Li-ion battery to 3 V, which can deliver high cycling stability and excellent energy density (Suo et al., 2015). The water-in-salt concept has been applied to other types of aqueous electrochemical energy storage systems, such as Na/Zn/K/Al-ion batteries and supercapacitors (Jiang et al., 2020; Zhao et al., 2016; Leonard et al., 2018; Zhou et al., 2019; Guo et al., 2019). The relatively lesser explored and overlooked area is the application of water-in-salt electrolytes for CIBs.

Based on the abovementioned considerations, we first proposed carbon material (graphene, carbon nanotubes, and carbon black) as cathode materials of CIBs and applied “water-in-salt” concept to widen the electrochemical window of chloride ion aqueous electrolytes. In this “water-in-salt” electrolyte system, the carbon cathode shows excellent cycling stability and electrochemical properties, and the discharge platform of battery is around 2.6V. Table 1 shows the comparison between our work and other representative CIBs. It can be clearly seen that the cycle life of CIB has highly improved compared with the previous chloride ion batteries. When graphene is used as cathode material, especially, the cycle life of CIB can be up to 2000. This provides useful insight into the development of high-performance CIBs, which has been retarded by the issue of electrode dissolution in electrolyte. The electrochemical reaction mechanism of the battery was investigated in detail by Transmission Electron Microscope (TEM), infrared spectroscopy (FTIR), X-ray photoelectron spectroscopy (XPS), and Raman spectra.

**Table 1:** Comparison of our work with traditional CIBs

Ref.	Cathode	Anode	Electrolyte	Voltage	Cycle life	Energy density (Wh/kg)
Zhao et al. (2014)	BiCl_3_	Li	OMIMCl-BMIMBF_4_	2.4V	3	181
Zhao et al. (2013)	FeOCl	Li	PP_14_Cl- PP_14_TFSI	2.1V	30	132
Zhao et al. (2017)	PPyCl@CNTs	Li	PP_14_Cl- PP_15_TFSI	2.2V	40	259
Gao et al. (2014)	VOCl	Mg	PP_14_Cl-PC	1.95V	53	90
Gao et al. (2016)	VOCl	Li	PP_14_Cl-PC	1.6V	100	184
Lakshmi et al. (2019)	Sb_4_O_5_Cl_2_	Li	PP_14_Cl-PC	1.5V	100	109
Yin et al. (2019)	CoFe-Cl	Li	PP_1_4Cl-PC	2V	100	280
Our work	Graphene	Zn	C_4_H_12_ClN-H_2_O	2.6V	2,000	252
	Carbon black			2.6V	1,000	194
	Carbon nanotubes			2.6V	800	205

Energy density equal to the product of C and U, where C is capacity based on the total mass of cathode-active material, U is average discharge voltage.

**Figure 1:**
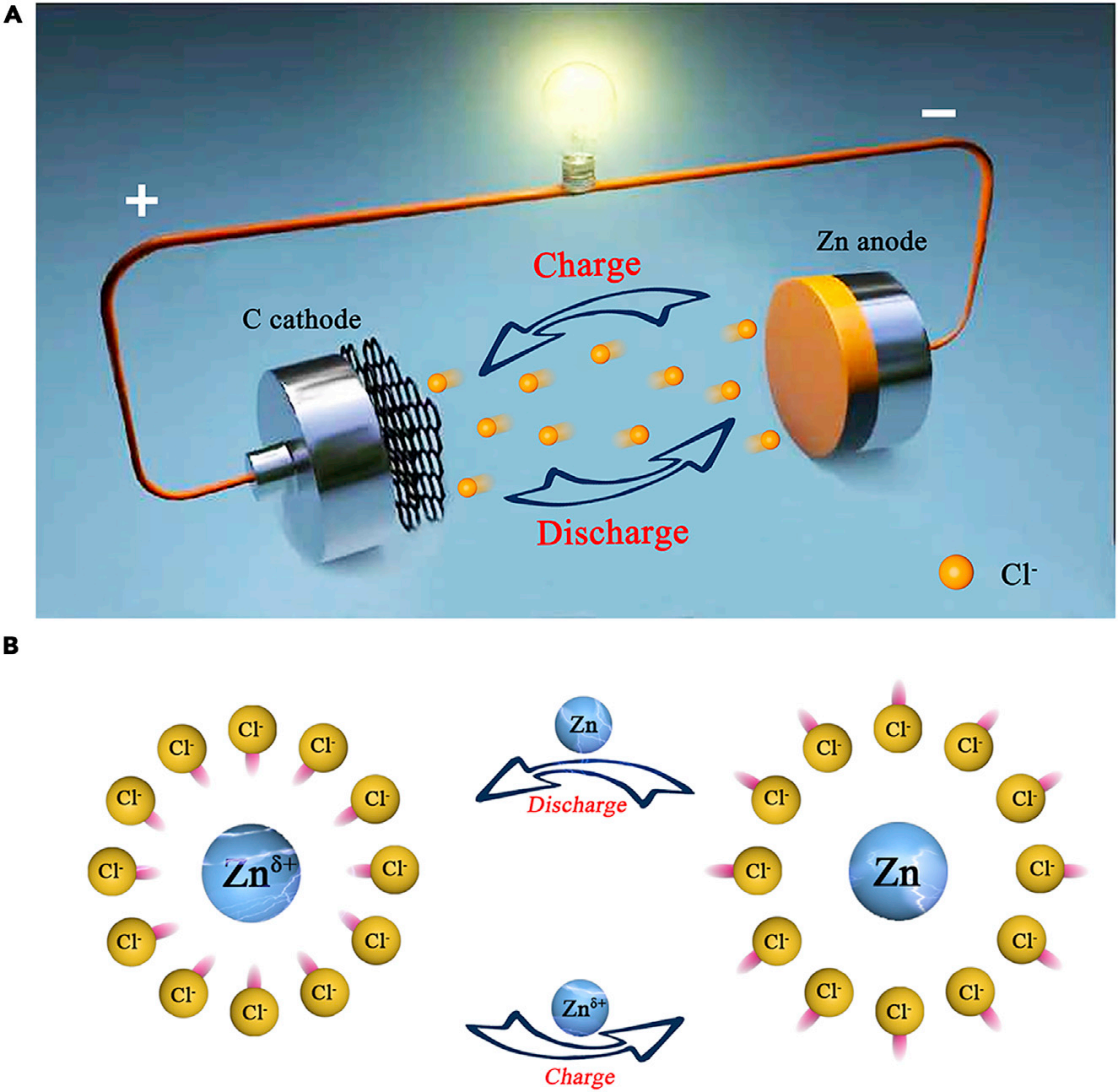
The reaction mechanism of CIB (A) Schematic representation of the rechargeable chloride ion battery. Herein, graphene, carbon black, and carbon nanotubes serve as cathode; Zn foil serves as cathode; and the saturated solutions of tetramethylammonium chloride was used as electrolyte. (B) The reaction mechanism of the negative electrode during the charging and discharging process.

The electrochemical reaction mechanism of the battery is based on the chloride ions shuttle via the absorption/desorption reactions of C/C_n_(Cl) at the cathode side. As shown in Figure 1A, during battery charge, the chloride ions desorb from metal anode and migrate to the carbon cathode. There, an absorption reaction occurs, leading to the formation of C_n_(Cl) phase (n is the molar ratio of carbon atoms to the absorbed Cl); during the discharging process, the chloride ions are desorbed from the carbon cathode and are captured by metal anode. For zinc anode (Figure 1B), during the battery discharge process, the chloride ions migrate to the zinc anode surface and the external electrons move from the anode to the cathode electrode in order to keep the balance of the internal circuit and external circuit. At the same time, the extranuclear electrons of zinc are pulled, but they have not completely separated from zinc, forming an intermediate state (Zn^δ+^) between Zn and Zn^2+^. At this time, there is an electrostatic attraction between the high concentration of chloride ions and the intermediate zinc, which makes the combination of chloride ions and Zn^δ+^ rely on weak intermolecular forces. During the battery charging progress, the electrons are reset, Zn^δ+^ becomes Zn, the electrostatic attraction disappears, and chloride ions desorb from the metal anode and migrate to the carbon cathode.

## Results and discussion

**Figure 2:**
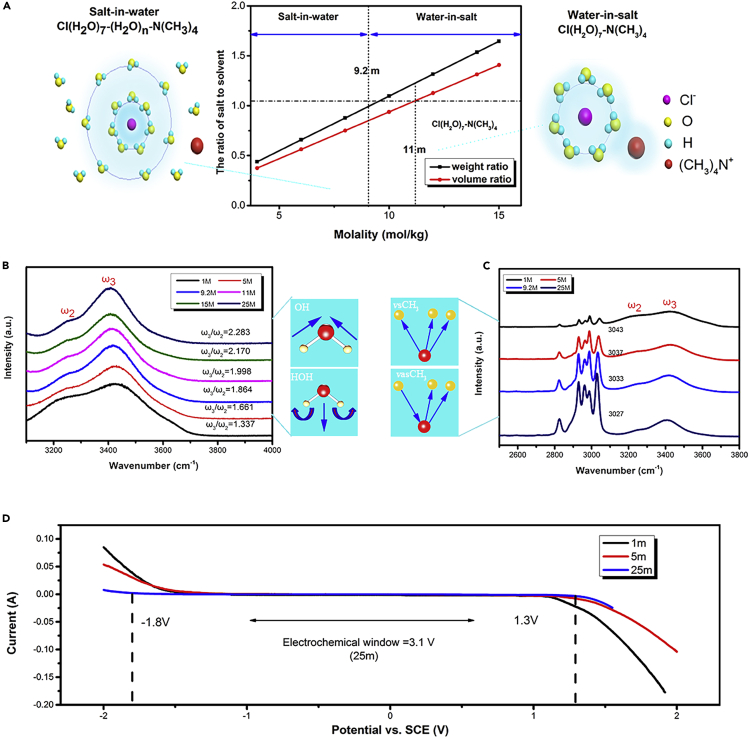
The water-in-salt electrolyte of CIB (A) The weight and volume ratio of ClN(CH_3_)_4_ to H_2_O change with increasing the molarity of ClN(CH_3_)_4_ in H2O. (B) Structure analyses of aqueous ClN(CH_3_)_4_ electrolytes. Comparison of Raman spectra of the O-H stretching vibration and HOH bending vibration of water molecules. (C) Comparison of Raman spectra of the CH_3_ stretching vibration of (CH_3_)_4_N^+^ anions. (Test parameters: 532 nm laser, 25%, 5 times objective lens, the time of one acquisition is 5 s, the average of the two acquisitions). (D) The electrochemical voltage window of the different concentrations (molality) ClN(CH_3_)_4_. Measurements were taken at different concentrations (molality) with linear sweep voltammetry (LSV) on graphite foil working electrodes between −2 V and 2 V versus Hg/Hg_2_Cl_2_ at 10 mV s^−1^.

As we know, water molecule has strong solvation power with its highly polar and high dielectric constant that the cations (anion) are often limited in solvation shells formed by O (H) atoms (Cresce et al., 2015; Bu et al., 2019). When the tetramethylammonium chloride concentration is below 9.2 m, the solvation sheath of Cl^−^ consists of at least two layers (Figure 2A). The first layer is a chemical hydration layer, in which water molecules and ions are firmly combined; the second layer is a physical hydration layer, in which the attraction of water molecules and ions is relatively weak, and the first Cl^−^ solvation shell typically contains seven water molecules (Bouazizi et al., 2006). However, when salt concentration is above 11 m, there are not enough water molecules available to form the “classical” primary solvation sheath; thus the resultant “water-in-salt” solution can then be visualized as a liquefied salt.

Figure 2B shows a comparison of the O-H stretching vibration and HOH bending vibration of water molecules in the different concentrated tetramethylammonium chloride electrolytes (Sun, 2009; Wu et al., 2017a; Frost et al., 2004). ω_2_ and ω_3_ represent the intensity of O-H stretching vibration and HOH bending vibration, respectively. It can be clearly seen that the O-H stretching vibration distinctly decreased with the consistence increase. The original shoulder at 3,248 cm^−1^ gradually decreased, and the peak at 3,406 cm^−1^ became the maximum at 25 m, showing that the strong hydrogen bonds of free water molecules were significantly destroyed. In addition, the ratio of ω_3_ to ω_2_ increased as the concentration increased, indicating that the destructive effect on the hydrogen bonds between water molecules is gradually increased as the concentration increases. The destruction of the hydrogen bonds reduces the activity of free water molecules, and hydrogen evolution reaction is not easy to occur during charging and discharging.

Figure 2C shows the evolution of the CH_3_ stretching vibration peak in the different concentrated tetramethylammonium chloride electrolytes. The bands at 3,027 cm^−1^ can be attributed to the asymmetric stretching vibration of CH_3_, and the two bands at 2,931 cm^−1^ and 2,990 cm^−1^ can be attributed to the symmetrical stretching vibration of CH_3_ (Ouasri et al., 2002; Mączka et al., 2004). The asymmetric stretching vibration of CH_3_ experiences a shift from 3,027 cm^−1^ to 3,043 cm^−1^ with the increasing concentration from 1 to 25 m, and the width of this band became narrow. This change can be attributed to the transition from ion pairs separated from water to contact ion pairs of Cl^−^ and (CH_3_)_4_N^+^ ions and the increasing order of electrolyte structure. This orderly electrolyte structure should inhibit the movement of water molecules, resulting in the reduced activity of free water molecules.

The electrochemical stability window for these aqueous electrolytes was evaluated with linear sweep voltammetry (LSV) on graphite foil electrodes, whose scans are shown in Figure 2D. The overall stability window extends with the increasing concentration of the ClN(CH_3_)_4_ electrolyte, with both hydrogen and oxygen deposition potentials far beyond the thermodynamic stability limits of water. Specifically, the electrochemical stability window for the 1 m ClN(CH_3_)_4_ electrolyte was 2.20 V (from −1.15 to 1.05 V versus SCE) and the electrochemical stability window for the 25 m ClN(CH_3_)_4_ WIS electrolyte was 3.10 V (from −1.8 to 1.3 V versus SCE), showing a 0.9 V expansion of the electrochemical stability window.

**Figure 3:**
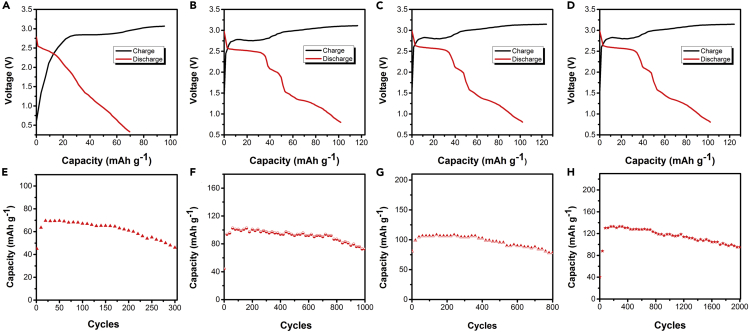
Electrochemical performance of chlorine-ion battery with different electrodes in the saturated solutions of tetramethylammonium chloride Typical charge-discharge voltage profiles at 1 A g^−1^ between 0.8 and 2.6 V of (A) carbon black/graphite, (B) carbon black/Zn, (C) carbon nanotube/Zn, and (D) graphene/Zn electrodes in the fifth cycle. Cycle life diagram of (E) carbon black/graphite, (F) carbon black/Zn, (G) carbon nanotube/Zn, and (H) graphene/Zn electrode.

The electrochemical performances of CIBs with different electrodes were investigated in assembled soft pack battery. Figure 3A shows the charge-discharge curves of the battery using carbon black as cathode and graphite foil as anode. The battery delivers a discharge capacity of 70 mAh g^−1^ (cut off 0.5V) and the open-circuit voltage at around 2.6 V. A discharged capacity of 46 mAh g^−1^ was observed after 300 charge-discharge cycles, with a capacity retention of 65.7% (Figure 3E). Such an extraordinary high-voltage feature can be attributed to the fact that the chlorine-water-in-salt electrolyte offers a 3.1 V window through suppressing hydrogen evolution on anode and reducing the overall electrochemical activity of water on cathode, providing a high-voltage feature.

Furthermore, it is feasible to explore a suitable anode material to improve the electrochemical performance of this battery. When zinc is used as anode, the electrochemical performance of battery with different cathode materials is shown in Figures 3B–3D and 3F–3H. As shown in Figures 3B and 3F, when carbon black was used as cathode, the reversible discharge capacity was 102 mAh g^−1^, and the discharge platforms were about 2.6 V and 2.1V. And the battery delivered a discharge capacity of 45 mAh g^−1^ in the first cycle with a gradual rise in capacity in the first few cycles, reaching the maximum value of 102 mAh g^−1^ at the 60th discharge followed by stable cycling afterward. The increase in capacity for the initial sixty cycles can be related to the activation of the electrodes. A discharged capacity of 72.4 mAh g^−1^ was observed after 1,000 charge-discharge cycles, with a capacity retention of 71%. It can be clearly seen that the battery shows a more stable discharge platform and a higher discharge capacity when zinc foil is used as the anode. Furthermore, the electrochemical performances of other cathodes (carbon nanotubes and graphene) were also investigated. Figure 3C shows the charge-discharge curves of the battery using carbon nanotubes as the cathode. The reversible discharge capacity was 108 mAh g^−1^, and two discharge platforms at around 2.6 V and 2.1 V were observed. The battery delivered a discharge capacity of 108 mAh g^−1^ in the initial cycles and retained at 77 mAh g^−1^ after 800 cycles, with a capacity retention of 71.3% (Figure 3G). Most importantly, when graphene was used as cathode, the battery delivers a reversible discharge capacity of 136 mAh g^−1^ (cut off 0.8V) and two discharge plateaus at around 2.6 V and 1.9 V (Figure 3D), and the Zn/C full cell delivered a reversible specific capacity of 95 mAh g^−1^ and a cycling life for up to 2000 cycles (Figure 3H). The superior cycling stability of graphene cathode probably due to graphene has high specific surface area (2,630 m^2^ g^−1^), which is higher than carbon nanotubes and carbon black and thus provided larger electroactive surface area in favor of the symmetrical distribution of the current density more evenly on the electrode surface. Furthermore, graphene structure allowed more chlorine ions transfer during battery charge and discharge process and enhanced electrolyte penetration to the electrode surface, resulting in excellent long-term stable cycling.

**Figure 4:**
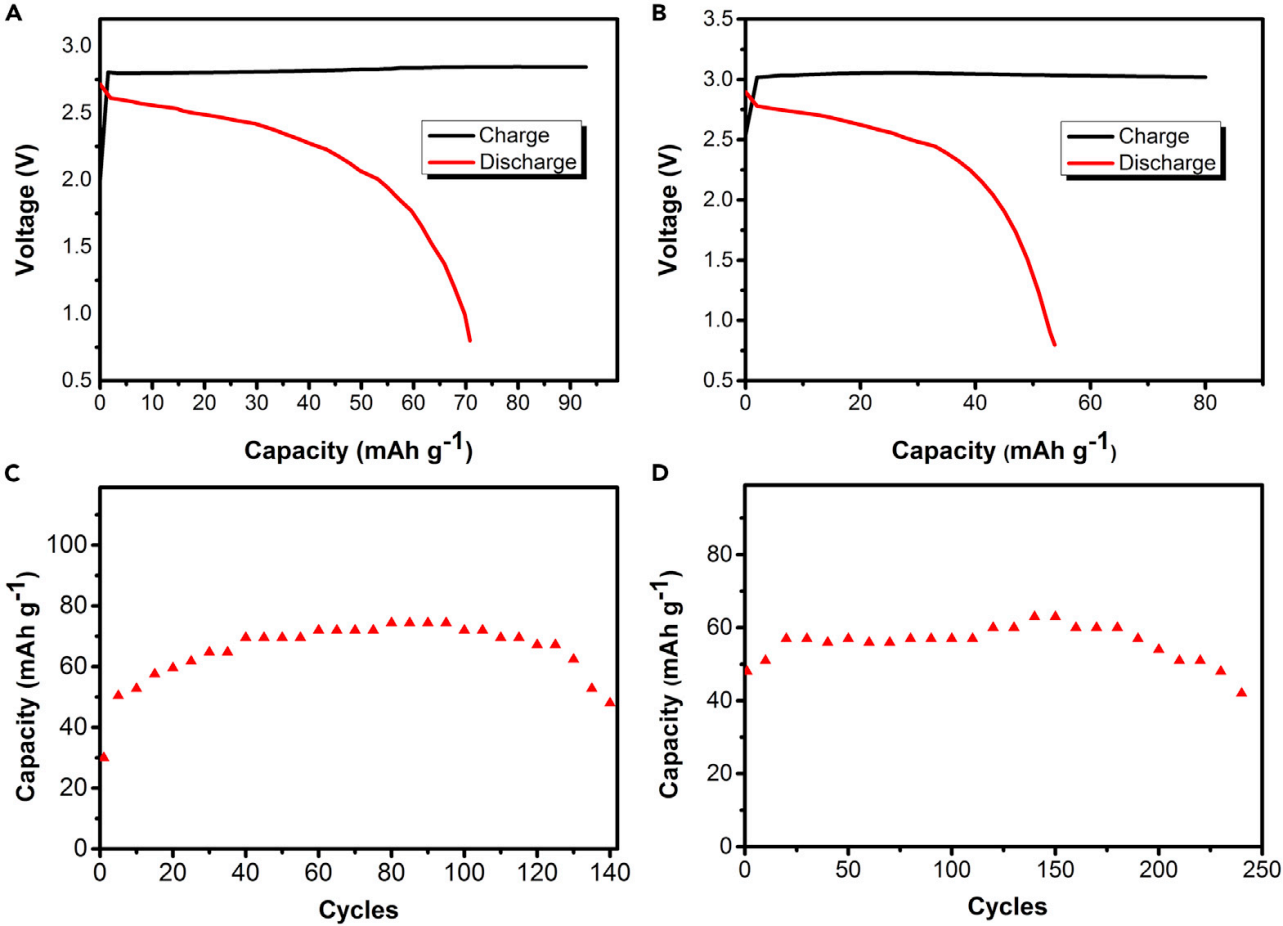
Performance of CIB with different metal anodes Typical galvanostatic charge/discharge curves for (A) tin foil and (B) aluminum foil as negative electrode at 1 A g^−1^ between 0.8 and 3 V of the cell when carbon black was used as the cathode in the fifth cycle. Cycle life diagram of (C) tin foil and (D) aluminum foil as anode of iodide-ion battery.

With the exception of zinc anode, we studied the cycle life and discharge performance of chlorine ion batteries with other anode materials in the saturated solutions of tetramethylammonium chloride. When tin foil was used as the anode, no discharge platform and a reversible capacity of 48 mAh g^−1^ was retained after 250 cycles as shown in Figures 4A and 4C. From Figures 4B and 4D, when aluminum foil was used as the anode, it can be seen that the battery delivered an operating voltage of about 2.3 V and a discharge capacity of 76 mAh g^−1^ in the initial cycles and retained at 49 mAh g^−1^ after 140 cycles, with a capacity retention of 65%.

**Figure 5:**
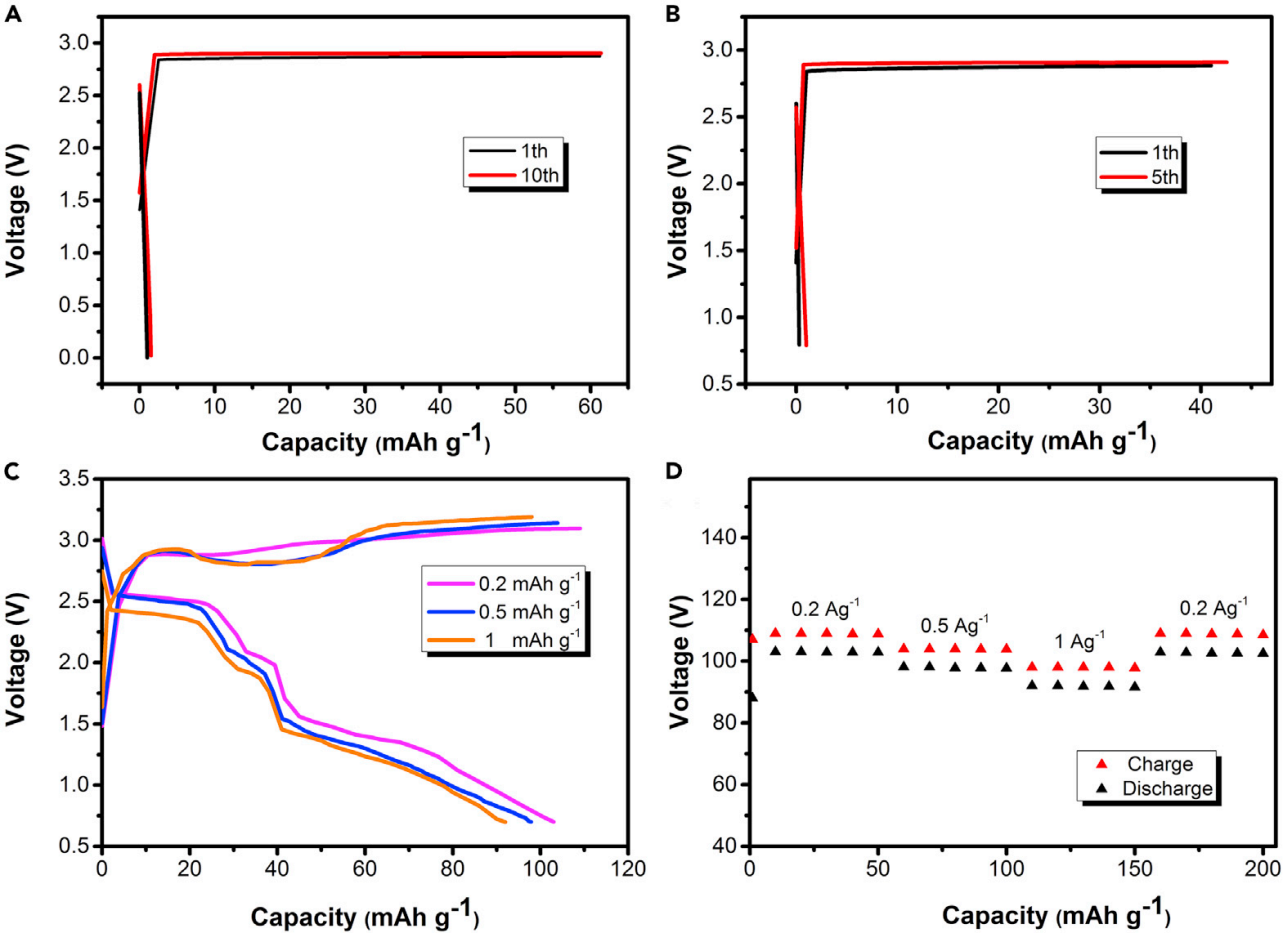
Performance of CIB Typical galvanostatic charge/discharge curves for (A) magnesium foil and (B) nickel foil as negative electrode at 1 A g^−1^. Potential profiles (C) and rate performance (D) of CIB at different current densities in the saturated solutions of tetramethylammonium chloride when carbon black and zinc foil were used as cathode and anode, respectively.

As shown in Figures 5A and 5B, magnesium foil and lead foil that were applied in previously reported CIBs were used as the anode. It can be seen that even with low current density discharge, there is no discharge platform and the specific capacity is close to zero, indicating metal magnesium and lead foil were not suitable for this electrochemical system. In short, although the cycling life of battery when tin foil and aluminum foil are used as negative electrodes is lower than the life of battery when zinc foil is used as negative electrode, it is still higher than previously reported CIBs. This result shows that a significant advantage of this new type of chloride ion battery is the possible use of abundant materials (Zn, Sn, and Al) as anodes.

Figures 5C and 5D shows the discharge/charge profiles of carbon black/Zn electrode at various current densities. The discharge capacities of 102 mAh g^−1^, 97 mAh g^−1^, and 92 mAh g^−1^ were obtained at 0.2 A g^−1^, 0.5 A g^−1^, and 1 A g^−1^, respectively. These results demonstrate that the cell is capable of delivering a high rate capacity and decent cycling stability.

**Figure 6:**
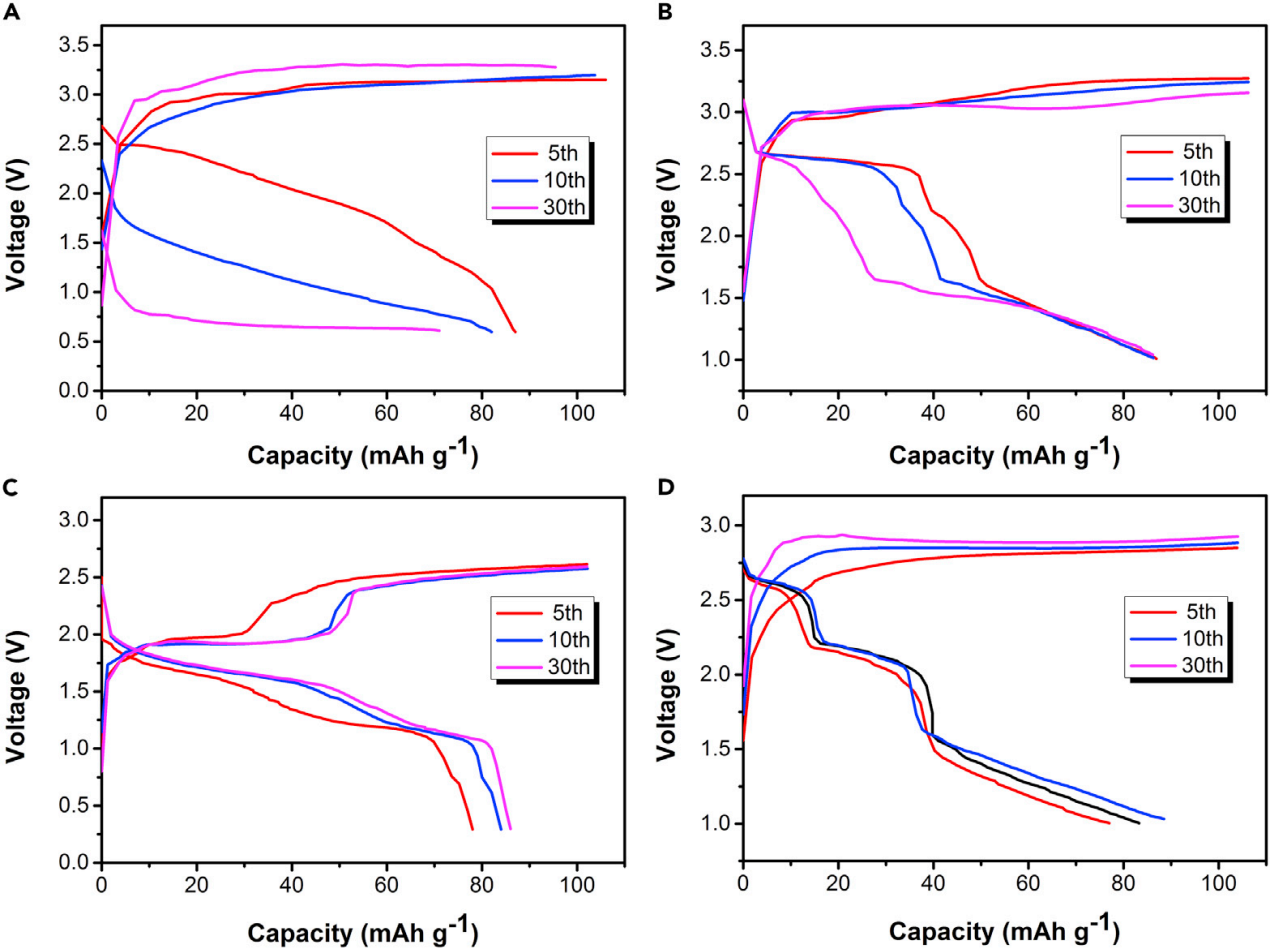
Charge-discharge voltage profiles of carbon black/Zn electrodes in the saturated solutions of tetramethylammonium chloride at the 5th, 10th, and 30th cycles when different cathode substrate is used (A) Stainless steel foil. (B) Chromium sheet. (C and D) (C) Nickel mesh and (D) Graphite foil.

Further, we studied the impact of different current collectors on battery performance. Figure 6A shows the charge-discharge voltage profiles when the stainless-steel foil is used as the current collector. The discharge capacity of the battery decreased sharply in the first thirty cycles; at the same time, the discharge platform of the battery decreased below 1V, showing an unstable state of the battery. When a chromium sheet was used as the current collector (Figure 6B), the discharge capacity of the battery has basically not decayed in the first thirty cycles, but its discharge platform is unstable. When nickel mesh is used as the current collector (Figure 6C), the battery maintains a stable state no matter what the charge-discharge platform or discharge capacity of the battery is. And when the graphite foil was used as the current collector (Figure 6D), the charge-discharge voltages and discharge platforms of the battery are in a best state compared with other current collectors. Therefore, graphite foil is the most ideal cathode substrate for chloride ion batteries.

**Figure 7:**
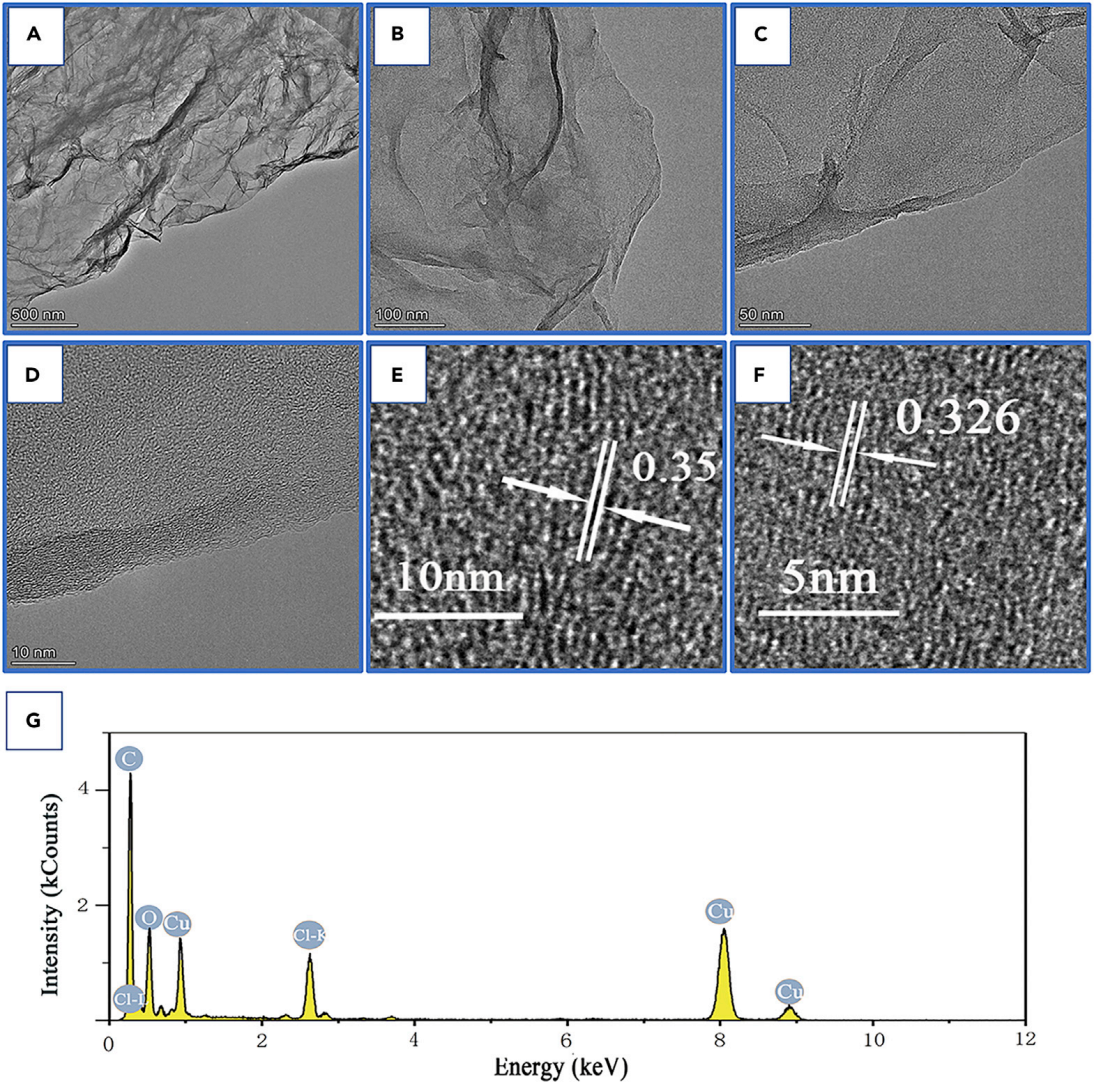
Microstructure characterization of graphene after the battery fully charged (A–C) TEM morphology images of graphene with differentscale bars. (A) 500 nm; (B) 100 nm; (C) 50 nm. (D) HRTEM images of graphene. (E) The HRTEM image of graphene, the spacing between the lattice fringe is 0.35 nm. (F) The HRTEM image of graphene, the spacing between the lattice fringe is 0.326 nm.(G) EDS images.

We performed transmission electronmicroscopy (TEM) experiments to characterize the microstructures of the graphene electrode after charging. The TEM images of the electrode are shown in Figure 7. From Figures 7A–7C, it can be clearly seen that there is a wrinkled structure in the edge layer of graphene, which may be caused by the absorption of chloride ions. Moreover, the lattice fringes on the surface of the graphene can be clearly observed in Figure 7D, where the lattice spacing of 0.326 nm (Figure 7F) and 0.35 nm (Figure 7E) corresponds to graphene and Cl-graphene, respectively (Zhao et al., 2015), indicating that the chloride ions absorbed to graphene after the battery fully charge. The corresponding EDS spectrum (Figure 7G) revealed the presence of C, O, and Cl, which imply that some OH groups in GO was substituted by chlorine, and the atomic percentage of C: Cl: O measured by EDS is 73.98:16.54:9.48 and the corresponding element weight ratio of C: Cl: O = 63.17:18.35:20.38.

**Figure 8:**
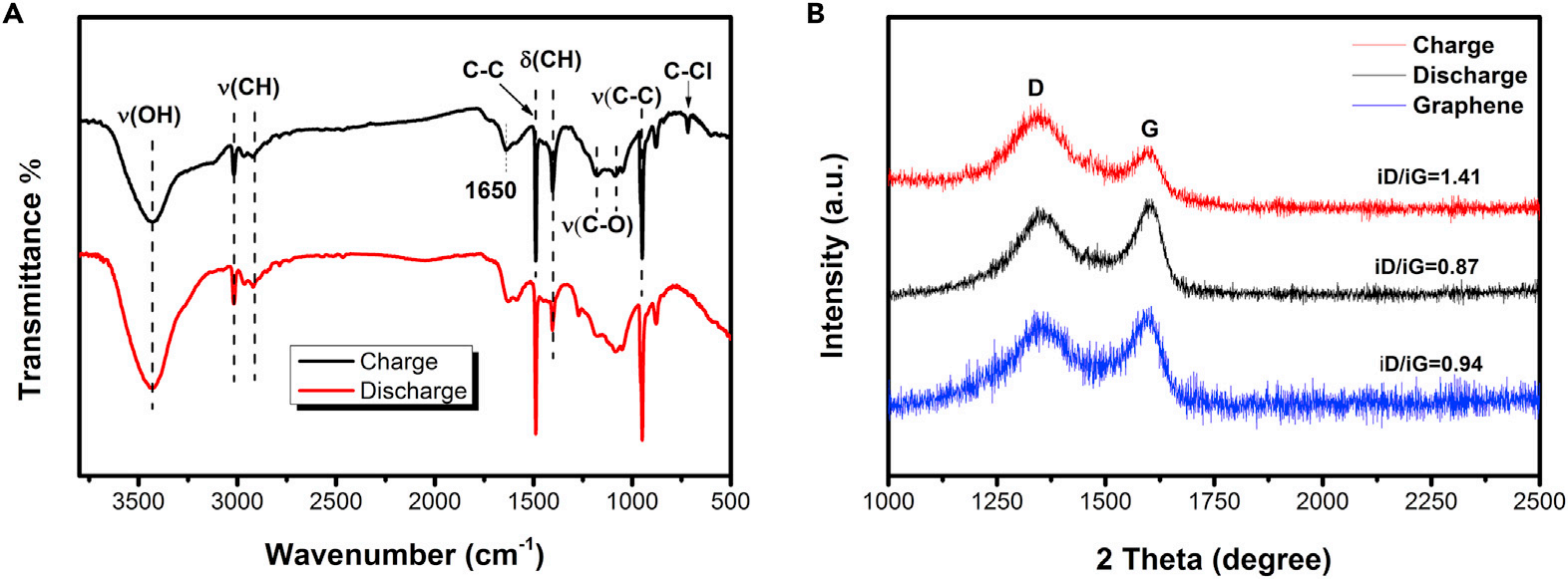
IR and Raman analysis of cathodes at various charge/discharge states (A) IR spectra of carbon black. (B) Raman spectrum of graphene.

The charge and discharge positive carbon materials were characterized by FT-IR and Raman to study the changes of the products during charge and discharge. As shown in Figure 8A, the bands at 3,430 (OH stretching vibration); 3,016 and 2,922 (C-H stretching vibration); 1,488 (C-C stretching); 942 (C-C out-of-plane vibration); and 1,340 (bending vibration) are observed in carbon black cathodes (Luo et al., 2014; Gussoni and Castiqlioni, 2000). When the charge is completed, the bands at 710 cm^−1^ can be clearly observed and attributed to the stretching vibration of C-Cl (Marcus, 2009). However, after the fully discharge, the stretching vibration of C-Cl at 710 cm^−1^ disappeared. This further confirms the chloride ions reversible absorption/desorption in the carbon cathode. In addition, two new peaks of stretching C-O at 1,088 and 1,185 cm^−1^ were also observed after fully charge (Cholpek et al., 2008), indicating that during the charging process, carbon atoms combined with oxygen atoms in the electrolyte and formed C-O bond. It is noteworthy that only weak intensity of C-Cl bands was observed due to the high absorption coefficient of carbon black in the IR region. Therefore, we further studied the Raman spectrum of graphene in different states. As shown in Figure 8B, there are two dominant peaks at 1,350 cm^−1^ and 1,600 cm^−1^ that correspond to the disordered sp3 hybrid carbon (D band) and crystalline sp2 hybrid structure (G band) in the Raman spectrum (Liu et al., 2015). The ratio of iD/iG indicates the defect degree of the graphene. When the battery fully charged, the ratio of iD/iG is 1.41, which is close to the iD/iG ratio of Cl-Graphene (Kakaei et al., 2016), indicating the existence of Cl-at edges or defects of the graphene during charging process. The iD/iG ratio of graphene decreased to 0.87 when the battery fully discharged, indicating its high-defect degree. It can attribute this difference to the effect of Cl^−^. The Cl^−^ ions deintercalate from the graphene to metal anode during the discharging process, contributing to the defect degree of the graphene increase.

**Figure 9:**
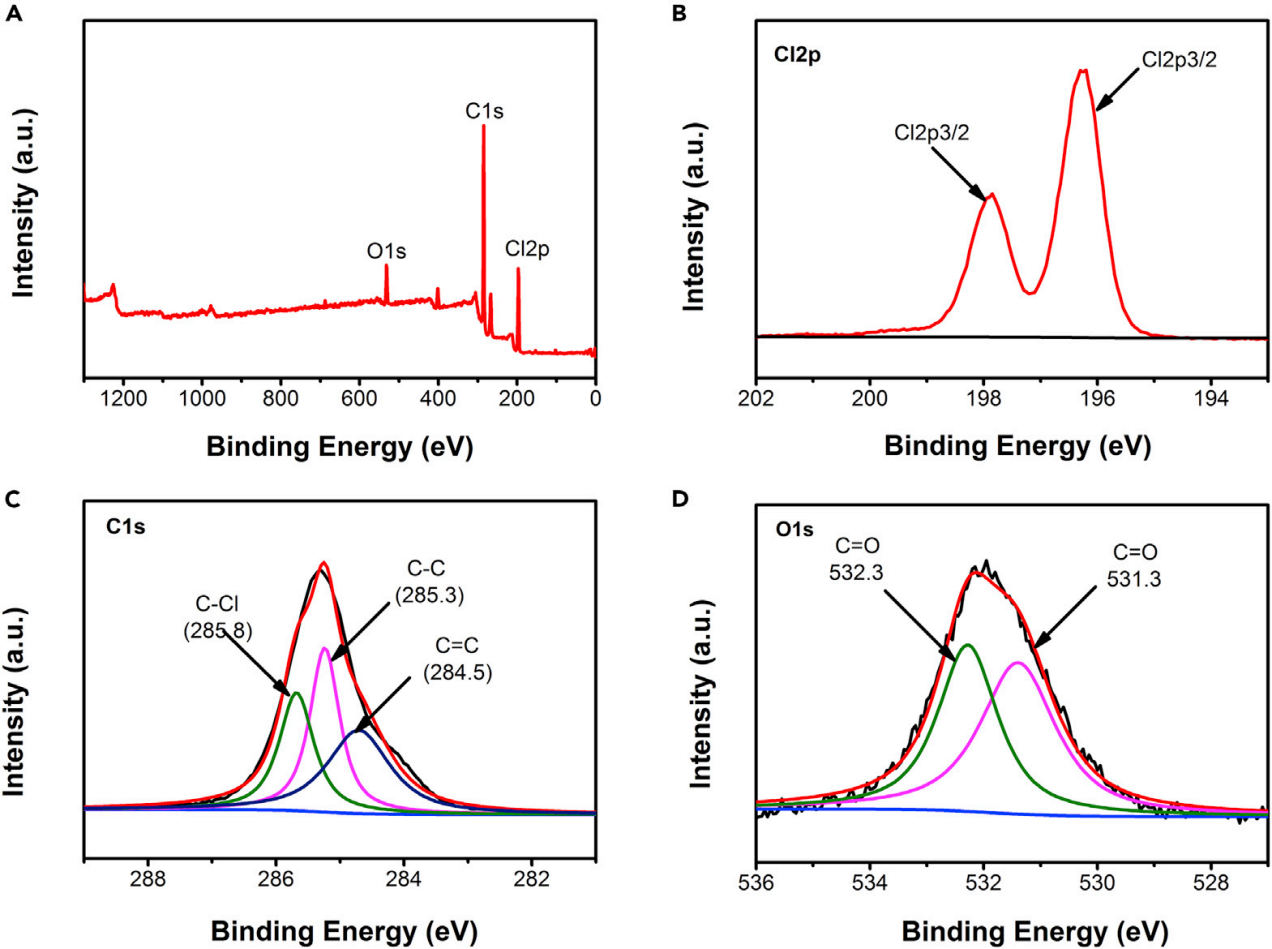
XPS spectra of carbon black cathode surface and elemental mapping images when the battery was fully charged (A) Carbon black surface region. (B) Cl 2p. (C) C1s. (D) O1s.

**Figure 10:**
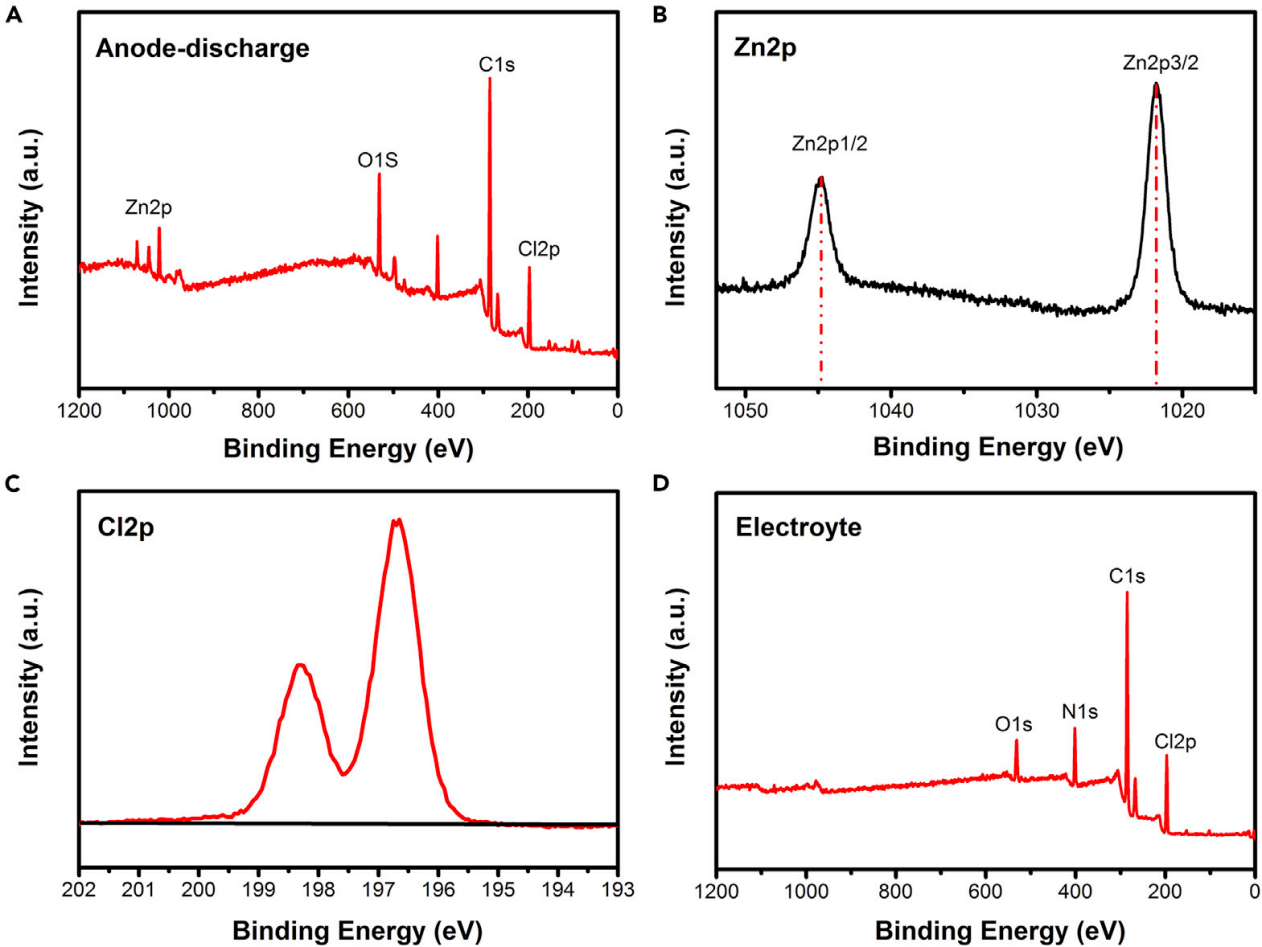
XPS spectra of Zn-anode surface and electrolyte (A–C) (A) The survey XPS spectrum of zinc anode and the spectrum of (B) Cl 2p and (C) Zn2p when the battery was fully discharged. (D) XPS region spectra of electrolyte.

Although the migration of chloride ions in the carbon cathode was confirmed by the analysis of FT-IR and XRD, zinc ion transfer in the CIB is not precluded owing to the zinc metal that was used as the anode, in which the electrochemical reaction mechanism also needs further explanation. Figures 9 and 10 show the X-ray photoelectron spectroscopy (XPS) spectrum for the carbon cathode, zinc anode, and electrolyte of the battery. The XPS spectrum of the cycled carbon black cathode is shown in Figures 9A–9D. Figure 9A shows the existence of Cl 2p, C1s, and O1s. As shown in Figure 9B, two peaks were observed in the Cl 2P region with binding energies of 197.8 eV and 196.2 eV, respectively, corresponding to Cl^−^ (Zhao et al., 2018; Yu et al., 2017). As showed in Figure 9C, the peaks of 284.5, 285.3, and 285.8 eV can be attributed to C=C, C-C, and C-Cl, respectively (Okpalugo et al., 2005; Golczak et al., 2008). The two peaks of Cl 2p and C-Cl indicated that Cl^−^ ions are inserted into the carbon and sufficiently combined with it in the charging process. In addition, the spectrum of O 1s is shown in Figure 9D. The peak of 531.3eV and 532.3eV can be attributed to C=O and indicates that carbon atoms form new chemical bonds during charging.

After discharge, the XPS spectrum of zinc anode is shown in Figure 10A. The peaks of Cl 2p at 198 (Cl 2p1/2) and 196.35 eV (Cl 2p3/2) can be clearly observed (Figure 10C). Those values are close to the binding energies of the Cl 2p of the carbon cathode when the battery is fully charged; therefore, no chemical change of chlorine can be evidenced, which remains as Cl^−^ ions, indicating that the s Cl^−^ ions deintercalate from the carbon cathode and are captured by metal anode during the battery discharge process. As shown in Figure 10B, two peaks of 1,021.8 and 1,044.8 eV are observed in the Zn 2p core level spectrum, indicating that Zn atoms are in an intermediate state of metal Zn^2+^ and zinc (Wu et al., 2017b; Naveed et al., 2019). For electrolyte, Figure 10D shows the existence of Cl 2p, C1s, N1s, and O1s without Zn signals, indicating that the zinc ion transfer is not included during the charge and discharge process. These results confirm the reversible transfer of chloride ions in the cathode and anode; zinc and chloride ions are bound together by intermolecular forces during discharging rather than dissolve/deposit in electrolyte.

**Figure 11:**
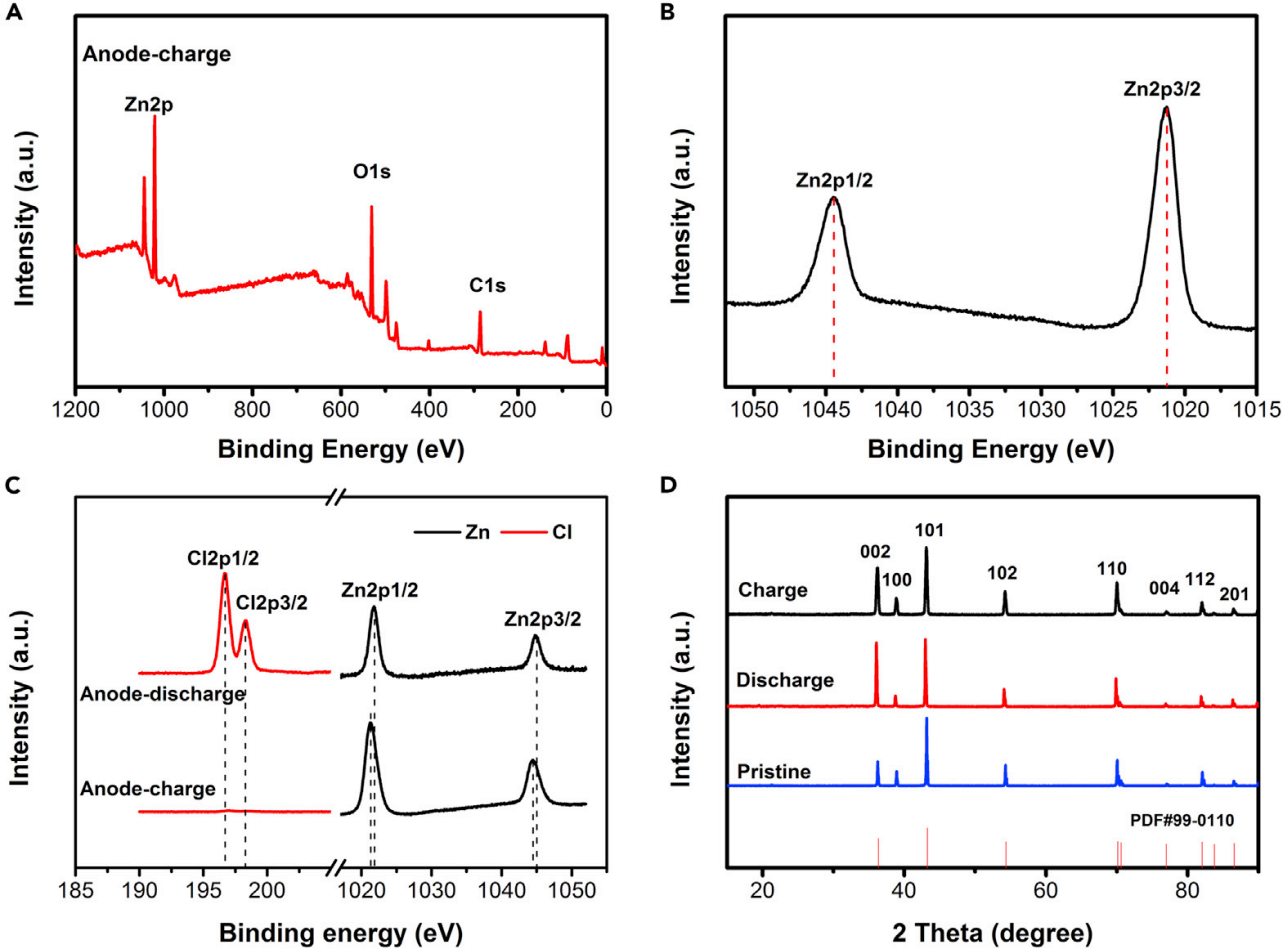
XPS and XRD spectra of Zn-anode at different electrochemical states (A and B) (A) The survey XPS spectrum of zinc anode and the spectrum of (B) Zn2p when the battery was fully charged. (C) XPS region spectra of zinc anode at the fully discharged and fully charged states. (D) XRD spectra of zinc anode when the CIB is fully charged and discharged.

Figures 11A and 11B show the XPS spectrum of zinc anode when the battery was fully charged. Figure 11A shows the existence of Zn 2p, C1s, and O1s. As shown in Figure 11B, two peaks were observed in the Zn 2P region with binding energies of 1,021.4 eV and 1,044.4 eV (Biesinger et al., 2010), respectively, corresponding to pure Zn. As shown in Figure 11C, after the discharge, the emergence of the Cl 2p peak doublet at 198 (Cl 2p3/2) and 196.35 eV (Cl 2p3/2) occurred. However, the chlorine element disappeared when the battery was fully charged, showing the chlorine transfer zinc anode to cathode during charging progress. The results of XPS show that Cl^−^ ions migrated to the zinc surface during the discharge process, but it is uncertain whether chemical bonds are formed. Therefore, we studied the XRD spectra of zinc anode when the CIB is fully charged and discharged (Figure 11D). Both charged and discharged, eight drum peaks of pure Zn at 2θ = 36.28°, 38.9°, 43.2°, 54.31°, 70.63°, 77.04°, 82.04°, and 86.53° can be clearly observed and correspond to the (002), (100), (101), (102), (110), (004), (112), and (201) directions of the Zn structure, respectively, and no new peaks were observed, confirming that zinc and Cl^−^ ions do not form a chemical bond; they are combined with electrostatic attraction during the battery discharge process.

### Conclusion

In summary, we proposed a CIB based on a carbon cathode, a metal anode, and the “water-in-salt” electrolyte. The significance of this work proposes new chloride ion storage electrode materials, finding a safe, economical, and high stability electrolyte that widens the electrochemical window of chloride ion aqueous electrolytes to 3.1 V, highly improving the cycle life compared to traditional CIBs. Through the application for battery testing, the graphene electrode delivers a stable capacity of 136 mAh g^−1^ (cut off 0.8V), and the cycle life can be up to 2000 cycles. Reversibility of chlorine ions absorption/desorption was confirmed by the analysis of TEM, XPS, FTIR, and XRD. This work provides a strategy for improving the discharge platform and cycle life of chlorine ion batteries. Meanwhile, CIB has high practical value and application prospect owing to its rich electrode material, simple preparation process, and efficient and safe discharge process in the field of energy storage.

### Limitations of the study

In this work, the chloride ions reversible absorption/desorption in carbon cathodes was confirmed by TEM, XPS, FTIR, and XRD, and graphene cathode shows better electrochemical performance compared with carbon nanotubes cathode and carbon black cathode. It is better to carry the *in situ* characterization into practice to analyze the role of graphene and understand the reaction mechanism more deeply.

### Resource availability

#### Lead contact

Further information and requests for resources and reagents should be directed to and will be fulfilled by the Lead Contact, Mingqiang Li (limingq@dlut.edu.cn).

#### Materials availability

This study did not generate new unique reagents.

#### Data and code availability

This study did not generate/analyze datasets/code.

## Methods

All methods can be found in the accompanying Transparent methods supplemental file.

## Supplemental information

Document S1. Transparent methods
