# Supplementary material for: High-voltage and long-lasting aqueous chlorine-ion battery by virtue of “water-in-salt” electrolyte
Source: iScience. 2021 Jan 5;24(1):101976. doi: 10.1016/j.isci.2020.101976 (PMC7797922; doi:10.1016/j.isci.2020.101976)
Supplement: Document S1. Transparent methods [file mmc1.pdf]

iScience, Volume *24*

Supplemental Information

High-voltage and long-lasting aqueous chlorine-ion battery by virtue of “water-in-salt” electrolyte

Tong Li, Mingqiang Li, Hang Li, and Hu Zhao

Transparent Methods

Material

Zinc foil, tin foil, aluminum foil, graphite foil, graphene, carbon nanotubes, carbon black, Polyvinylidene fluoride (PVDF) , and N-Methyl pyrrolidone (NMP) were purchased from Aladdin reagent official website. Tetramethylammonium chloride was purchased from Tianjin Damao Chemical Reagent Factory.

Preparation of C electrode

Carbon materials (carbon black, carbon nanotubes, graphene) and PVDF with a mass ratio of 8:2 were weighed. Then the mixture is added to the solvent of N-methyl-2-pyrrolidone to form a uniform slurry, which was spread evenly on a graphite foil (2.5 cm×2.5 cm), and then was dried in a vacuum oven at 70 °C for 5 h.

Electrochemical measurements

Electrochemical measurements were performed with soft pack battery. The full cells were assembled by using the prepared C carbon electrode (2.5 cm×2.5 cm) as the cathode, a zinc metal foil as the anode, a polyolefin microporous membrane as separator, and the saturated solutions of tetramethylammonium chloride was used as electrolyte. The galvanostatic charge-discharge tests were conducted on a BTS4000 battery program-control test system at room temperature. The discharged capacity of the battery is based on the the total mass of cathode active material. The three-electrode devices for cathode consists of carbon rod as working, Pt as the counter and $Hg/HgCl_2$ as the reference electrode, respectively.

Material characterization

Powder XRD patterns were collected on a X-ray diffractometer (D/Max 2400, Japan) with Cu Kα radiation (λ = 0.15406 nm). Fourier transform infrared spectroscopy (FTIR) spectrum was recorded with a NICOLET 6700 FT-IR Spectrometer using KBr pellets. XPS was tested on a Thermo ESCALAB XI^+ equipped with a hemispherical analyzer. The microstructure and morphology of graphene was characterized by a transmission electron microscope (TEM, JEM-1200EX 120KV).
